# Utilisation of cardiopulmonary exercise testing for tailored pulmonary rehabilitation in people with interstitial lung diseases: A systematic review

**DOI:** 10.1177/02692155251391661

**Published:** 2025-10-30

**Authors:** Ben Bowhay, Craig A Williams, Sophie Goodrum, Tom Lacy-Kerr, Michael A Gibbons, Chris J Scotton, Owen W Tomlinson

**Affiliations:** 1 Department of Clinical and Biomedical Sciences, Faculty of Health and Life Sciences, 3286 University of Exeter, Exeter, UK; 2 Academic Department of Respiratory Medicine, Royal Devon University Healthcare NHS Foundation Trust, Exeter, UK; 3 NIHR Exeter Biomedical Research Centre, University of Exeter, Exeter, UK; 4 Department of Public Health and Sport Sciences, Faculty of Health and Life Sciences, University of Exeter, Exeter, UK

**Keywords:** Physiotherapy, rehabilitation, physical fitness, respiratory, PRISMA

## Abstract

**Objective:**

To systematically evaluate the effects of cardiopulmonary exercise test-derived, tailored pulmonary rehabilitation on cardiopulmonary outcomes in individuals with interstitial lung diseases.

**Data sources:**

MEDLINE, Embase, CINAHL Ultimate, SPORTDiscus, CENTRAL, and the Cochrane Library were searched from inception up to 4th September 2025. Reference lists of the included studies were hand-searched for additional sources.

**Review methods:**

Reporting followed PRISMA 2020 guidelines. Studies of any design published in English and involving participants with interstitial lung disease were eligible. Due to intervention heterogeneity, meta-analysis was not conducted.

**Results:**

Eleven studies comprising 321 participants were included, with sample sizes ranging from 1 to 52. Designs encompassed single-cohort interventions (*n* = 4), comparative interventional studies (*n* = 3), randomised controlled trials (*n* = 3), and one case report. Pulmonary rehabilitation interventions included aerobic, interval, and resistance training, delivered over study durations ranging from 4 weeks to 4.5 years. Cardiopulmonary exercise testing outcomes included peak oxygen uptake; peak work rate; peak minute ventilation; maximum heart rate, and rate of perceived exertion. All studies assessing peak oxygen uptake and peak work rate reported improvements. Peak minute ventilation improvements were reported in six of seven studies. No serious adverse events were reported.

**Conclusion:**

Tailored pulmonary rehabilitation via cardiopulmonary exercise test metrics appears to enhance peak oxygen uptake and peak work rate in individuals with interstitial lung disease. Findings support its potential efficacy; however, future research should prioritise standardised methods, consistent reporting, and longer follow-up durations to inform clinical practice.

## Introduction

Interstitial lung diseases are a heterogeneous group of ∼200 chronic lung conditions, which are associated with lung parenchymal fibrosis and/or interstitial inflammation.^1^ Unfortunately, these chronic conditions cannot be cured, and people with idiopathic pulmonary fibrosis, one of the most common subtypes of interstitial lung disease, have a median survival of 2–3 years from diagnosis.^2^ Therefore, maintaining lung function, physical activity and quality of life for people with interstitial lung disease is a key focus.^3^

A joint statement produced by the American Thoracic Society and European Respiratory Society recommends pulmonary rehabilitation to enhance cardiorespiratory health and quality of life outcomes for people with interstitial lung diseases.^4^ Thus, the National Health Service, alongside the National Respiratory Audit Programme aim to improve pulmonary rehabilitation services in the United Kingdom.^5^ The 2023 British Thoracic Society ‘Clinical Statement on Pulmonary Rehabilitation’ states validated exercise tests should be conducted to inform tailored exercise prescription.^6^ A systematic review from Barratt et al.^7^ highlighted the value of cardiopulmonary exercise testing (CPET) within interstitial lung disease care. Moreover, CPET is valid and reproducible in interstitial lung disease,^8,9^ supporting clinical implementation.

CPET is the gold standard for assessing exercise intolerance in chronic pulmonary conditions.^10^ It provides integrated evaluation of cardiac, respiratory, and muscular function, and identifies limiting factors such as breathlessness and low motivation.^11,12^ In clinical settings, CPET can quantify aerobic capacity^13–15^ and guide tailored exercise prescription.^16,17^ Despite established pulmonary rehabilitation guidance in respiratory diseases,^18^ no frameworks exist for interstitial lung diseases. Therefore, the James Lind Alliance ranked exercise optimisation for pulmonary fibrosis a top 10 research priority.^19^

The challenge is exercise tolerance has been shown to be markedly reduced in interstitial lung diseases,^20^ compounded by multisystem pathophysiological impairments,^11^ which impacts the applicability of generic pulmonary rehabilitation.^21^ Furthermore, field tests such as the 6-min walk distance and incremental shuttle walk test may not provide the physiological detail needed to tailor exercise in this population.^15^ In contrast, CPET quantifies real-time physiological responses, enabling threshold-based exercise prescription using metrics like maximal heart rate, peak oxygen uptake, and peak work rate.^11,22^

To date, no systematic reviews have evaluated how CPET-derived measures can guide personalised rehabilitation for interstitial lung diseases. This review addresses that gap, identifying relevant parameters and intensities, and assessing their impact on cardiorespiratory health.

## Methods

This systematic review was conducted and reported using the updated PRISMA 2020 guideline.^23^ The protocol was registered on PROSPERO (CRD: 42024543174) on 7th May 2024, and a full protocol had published previously.^24^ All full-text articles were obtained and screened for eligibility against the inclusion and exclusion criteria represented in Table 1.

**Table 1.** Inclusion and exclusion criteria.

Category	Inclusion criteria	Exclusion criteria
Population(s)	ILD patients of all ages, genders and disease severities.	Non-ILD patients.
Intervention(s)	Tailored pulmonary rehabilitation or exercise programmes. Exercise prescribed by using variables derived from cardiopulmonary exercise testing.	Articles which only describe ‘physical activity’ interventions and not ‘exercise training’ or ‘pulmonary rehabilitation’ will be excluded. Single bouts of exercise do not classify as exercise training in this context.
Comparators	Control where possible.	Not specified.
Outcomes	Peak volume of oxygen consumption (VO_2peak_); peak work rate (WR_peak_); maximum heart rate (HR_max_); peak ventilation (VE_peak_).	Outcomes which do not meet inclusion criteria outcomes.
Study design	Full text studies. Published in English. No restriction on publication date, or location.	Articles which are not published in English, unless an English translation is available.
Setting	All settings.	Nil.

ILD: interstitial lung disease; VO_2peak_: peak volume of oxygen consumption; WR_peak_: peak work rate; HR_max_: maximum heart rate; VE_peak_: peak ventilation.

Electronic searches were conducted using Ovid^®^, incorporating Ovid MEDLINE^®^ and Ovid Embase™, EBSCO databases CINAHL Ultimate and SPORTDiscus, CENTRAL and the Cochrane Library. Searches were conducted in two phases, firstly from inception up to January 2025, and secondly from January 2025 to September 2025 as an update following initial peer review. The reference lists of the included studies and review articles were also reviewed to identify any potentially relevant studies not identified through the search process. No grey literature, theses, or dissertations were included. The full strategy is available online.^25^

Initial search results obtained using the search strategy described above were screened for eligibility and inclusion according to title and abstract. Independent data extraction was performed using customised Microsoft Excel™ data extraction forms developed specifically for the study. The search results in the first search were double-screened by BB and TL-K, with OWT as a third reviewer if needed. In the second post-review search update, these were double-screened by BB and OWT.

Quality assessment of the included studies was performed by two reviewers (BB and SG) using the critical appraisal skills programme checklists for single-cohort studies^26^ and randomised controlled studies.^27^ A third reviewer (OWT) was available if needed. The critical appraisal skills programme checklist items were assigned a numerical value of ‘1’ for ‘Yes’, if well described, and a value of ‘0’ for ‘Can’t Tell’ and ‘No’, if inadequate.^28,29^ The consensus on exercise reporting template^30^ was utilised to assess the reporting and delivery of exercise interventions within the included studies. Assessment was performed by two reviewers (BB and SG), with a third reviewer (OWT) available if required.

The results of the included studies were synthesised based on the reported interventions and outcomes. Meta-analysis was not performed due to the wide heterogeneity of study design and interventions within the included studies, but also the lack of standardisation in outcomes. Therefore, the results are narratively synthesised based on the key findings of the outcome measures reported in the included studies.

There were some deviations from the published protocol to note. Firstly, the screening of all the included papers was due to be independent, with 10% double-screened by another member of the research team. However, it was decided to perform full double screening to enhance accuracy and reduce bias. Secondly, meta-analysis and assessment of evidence certainty were not performed due to widespread heterogeneity within the included studies. Thirdly, as formal data analyses were not performed, use of Review Manager^30^ was not necessary. Outcome measures detailed in the protocol (e.g. volume of oxygen consumption at anaerobic threshold and rate of perceived exertion) were not detailed in main text, but are available in the Supplementary materials hosted on Open Science Framework.^25^

## Results

### Study selection and characteristics

**Figure 1.**
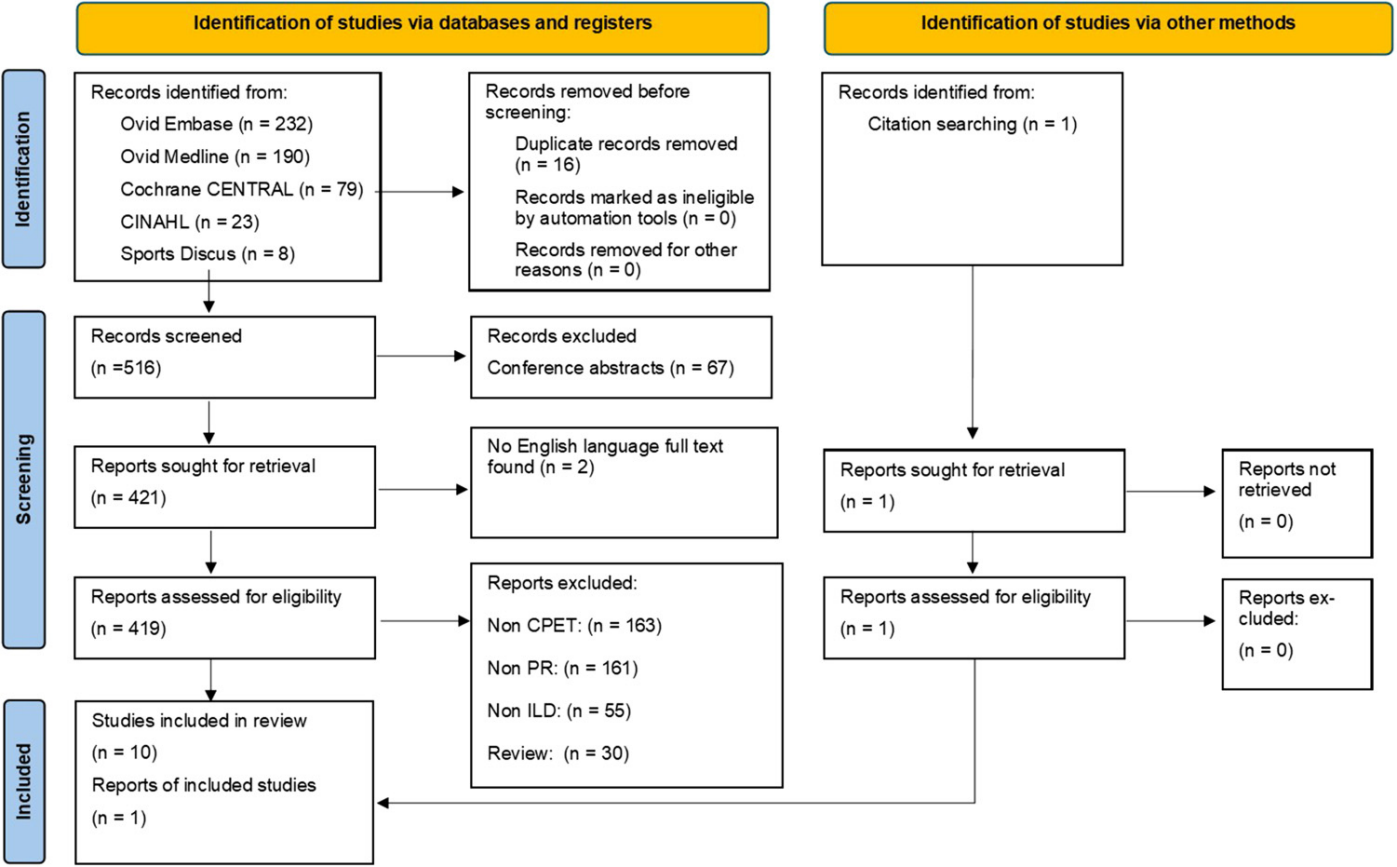
PRISMA flow chart. CINAHL: Cumulative Index to Nursing and Allied Health Literature; CPET: cardiopulmonary exercise test; PR: pulmonary rehabilitation; ILD: interstitial lung disease.

Electronic and hand searches identified 532 studies were suitable for inclusion screening. After assessing the articles against the inclusion criteria, 11 studies were included in the review (Figure 1), following good agreement (82%) in the initial double-screening process by BB and TL-K, before consensus (100%) was achieved via discussion. No additional articles were identified for inclusion in the second search phase, with 100% agreement between BB and OWT. Reasons for article exclusion are detailed in the Supplementary materials on Open Science Framework^25^ and the selection process has been reported using a PRISMA flow diagram.

A summary of the included study characteristics is presented in Table 2. Study designs included four single-cohort intervention studies,^31–34^ three comparative interventional studies,^35–37^ three randomised controlled trials,^38–40^ and one case report.^41^ Four studies did not use a control group.^31,32,34,41^ A control group of continued medical management was utilised in six studies.^35–40^ One study used an age- and sex-matched cohort of participants without a diagnosis of idiopathic pulmonary fibrosis as a control.^33^ A control group consisting of study drop outs was formed in one study.^40^

**Table 2.** Study and participant characteristics.

Authors and study design	Participant demographics	Intervention	Comparison	Outcomes and follow-up
Arizono et al. (2014) Prospective observational study: PR vs control Cycle CPET	*n* = 24 Age: 69.4 ± 7.4 years Gender = 16 M; 8F IPF diagnosed	Duration: 10 weeks Frequency: 2× per week Intensity: 80% of peak work rate (target) obtained from an incremental load ergometry exercise test. 30% of maximum inspiratory pressure during resistance breathing training Time: 90-min sessions: 20 min of continuous cycling; 15 min breathing training. The remaining time was allocated to respiratory care, subject education, endurance training, and strength training. Type: Peripheral muscle strength training included upper and lower limb resistance training with weight machines, hand weights, or elastic bands. Respiratory muscle training.	Control: non-PR sample analysed: *n* = 27	Outcomes: VO_2max_; WR_peak_ Follow-up: no follow-up period Drop-outs: *n* = 5
Dale et al. (2014) Randomised controlled trial Cycle CPET	*n* = 35 Age: 70 ± 7 years Gender = 35 M, 0F ILD diagnosed; (Asbestos-related pleural disease, ARPD): *n* = 24; Asbestosis: *n* = 4; silicosis: *n* = 3, combined ARPD and asbestosis: *n* = 2; mixed-dust pneumoconiosis: *n* = 1)	Duration: 8 weeks The training mode, intensity, and duration were based on the recommended guidelines for COPD. Frequency: 3× per week Intensity: Cycle training 60% of peak work rate achieved on the incremental cycle test. Walking training: 80% of the average walking speed of the better baseline 6MWT. Time: 15 min walking and 15 min cycling at each session in the first training week. Progressing to 30 min of each modality by the final week. Type: Aerobic exercise training (walk and cycle)	Control: no exercise intervention – continued usual medical management Sample analysed: *n* = 17	Outcomes: VO_2max_; HR_max_; WR_peak_; VE_peak_ Follow-up: 26 weeks Drop-outs: *n* = 2
Vainshelboim et al. (2014) Randomised controlled trial Cycle CPET	*n* = 32 Age: 68 ± 8 years Gender = 21 M, 11F IPF diagnosed	Duration: 12 weeks Frequency: 2× per week Intensity: Block 1: cycling was set at 50–60% of peak WR achieved during the CPET, and 70–80% of individual average walking speed measured during the 6MWT. In addition, during the first sessions the workload was adjusted individually using the Borg CR-10 scale on the level of 3–5. Block 2: Increased up to 60–70% of WR_peak_ in cycling and 80–90% of individual average walking speed for treadmill walking and corridor walking. Time: 60-min group exercise per session Type: Aerobic interval training (treadmill walking, leg-cycling, and step climbing = 30 min. Resistance training with dumbbells (upper and lower body) = 10 min. Warm up: calisthenics, short active stretching, and deep breathing exercises = 5 min. Flexibility training = 5 min. Block 2: Interval training replaced with continuous aerobic endurance training = 20 min	Control: standard care Sample analysed: *n* = 17	Outcomes: VO_2max_; HR_max_; WR_peak_; VE_peak_ Follow-up: no follow-up period Drop-outs: *n* = 2
Keyser et al. (2015) Single-cohort intervention study Treadmill CPET	*n* = 13 Age: 42–69 years Gender = 5 M, 8F ILD diagnosed	Duration: 10 weeks Frequency: 3× per week Intensity: Target 70–80% of the heart rate reserve Time: 30–45 min per session Type: Aerobic exercise training sessions	No comparators. Sample analysed: *n* = 13	Outcomes: VO_2max_; HR_max_; WR_peak_; VE_peak_ Follow-up: no follow-up period Drop-outs: *n* = 0
Grongstad et al. (2020) Single-cohort intervention study Treadmill CPET	*n* = 41 Age: 53 ± 11 years Gender = 20 M; 21F Dropouts not included (*n* = 2) Sarcoidosis diagnosed	Duration: 4 weeks Frequency: Standard activity plan: 2–4× per week; Treadmill HIIT or high-intensity resistance training: 2–3× per week Intensity: High-intensity endurance interval training programme (4 × 3 min intervals on a treadmill at 85% of maximal heart rate; High-intensity resistance training (4 sets of 5 repetition maximum). Time: 45-min per session Type: High intensity resistance and endurance training	No comparators	Outcomes: VO_2max_; HR_max_; VE_peak_ Follow-up: no follow-up period Drop-outs: *n* = 2
Herrera-Olivares et al. (2020) Case report Cycle CPET	*n* = 1 Age: 52 years Gender = 0 M; 1F Severe Sarcoidosis diagnosed	Duration: 4.5 years Frequency-Intensity-Time-Type principles: Please refer to the publication main text for full details. This is due to the depth of information available, and large variability made throughout the 4.5 year study duration.	No comparators	Outcomes: VO_2max_; HR_max_; WR_peak_; VE_peak_; Follow-up: 4.5 years total Drop-outs: *n* = 0
Guber et al. (2021) Prospective, interventional before/after study Cycle CPET	*n* = 52 Age: 53.5 ± 11 years Gender = 27 M; 25F Sarcoidosis diagnosed	Duration: 12 weeks Frequency: 2× per week Intensity: Moderate intensity, 70% HR_max_, as calculated by the Karvonen formula and by the anaerobic threshold on CPET. Target intensity was set at 12–14 on the Borg RPE scale - considered a moderate level. Time: 90 min Type: 20 min of balance, weight, flexibility, and range of motion exercises; 40 min of aerobic exercises on a variety of ergometers (10–15 min each); 30 min of strength exercises on 11 major muscles groups (2–3 sets of 10–15 repeats/min each).	Control: non-PR, same cohort analysed: *n* = 52	Outcomes: VO_2max_; HR_max_ Follow-up: 6 months Drop-outs: *n* = 14 at 6 month follow-up
Essam et al. (2022) Randomised trial Cycle CPET	LLEG *n* = 10 ULEG *n* = 10 Age: LLEG = 44.40 ± 12.25 years ULEG = 41.90 ± 7.58 years Gender: LLEG = 6 M, 4F ULEG =2 M, 8F ILD diagnosed	Duration: 6 weeks Frequency: 3× per week supervised Intensity: Measured as the percentage of the maximum heart rate determined from the equation (220 − age of the participant). Lower limb aerobic training was performed on a treadmill Time and type: LLEG: Week 1: Treadmill low intensity exercise i.e. 50–60% of their maximum heart rate, duration of 10 min that was broken into shorter intervals if needed (as cycles of 3 min training followed by 1–2 min of rest period). Week 2: Increased duration of session by 5 min every 2 sessions, with decreasing the intervals between training, and increasing the work- load by 5% every 2–3 sessions according to patient's tolerance. Week 3: 30 min of aerobic exercise: 2 cycles of continuous 15 min aerobic training separated by one interval of rest at moderate exercise intensity of 64–76% of their maximum heart rate. Weeks 4–6: 30 min continuously at moderate exercise intensity. Time and type: ULEG: Upper limb exercise was performed on a wheel. 15 min of continuous exercise plus incentive spirometer breathing exercises.	Control. Sample analysed: Control = dropouts (*n* = 11)	Outcomes: VO_2max_; HR_max_; WR_peak_; VE_peak_ Follow-up: unclear Drop-outs: *n* = 11.
Child et al. (2023) Single-cohort intervention study Treadmill CPET	*n* = 15 Age: 36.6–59.3 years Gender = 15F LAM diagnosed	Duration: 12 weeks Frequency: 4 days/week Intensity: Steady-state intensity exercise at 65–75% HR reserve, using the patients’ resting and peak HR observed at baseline maximal CPET. Time: Workout duration initiated at 25 min Gradually increased to 45 min by the end of week 2. Type: aerobic + body weight-resistance exercise training occurred 3 days/week. During exercise, participants were instructed to maintain SpO_2_ ≥ 85%, to monitor for symptoms of exercise intolerance, and to adjust exercise intensity and supplemental oxygen accordingly.	No comparators Sample analysed: *n* = 15	Outcomes: VO_2max_; HR_max_; WR_peak_; VE_peak_ Follow-up: no follow-up period Drop-outs: *n* = 1
Choi et al. (2023) Prospective, interventional, comparative study Treadmill CPET	*n* = 25 Age: 68 ± 5.5 years Gender = 23 M; 2F IPF diagnosed	Duration: 8 weeks Frequency: 3× per week Intensity: 10-min warm up at 50–70% HRR, followed by five 3-min intervals at 70–85% HRR separated by four 3-min active pauses at 50–70% HRR; 10-min cool down at 50–70% HRR Time: 10 min: Breathing retraining and chest expansion exercise; 47 min: Aerobic exercise on treadmill; 10 min: Resistance exercise Type: Breathing retraining included diaphragmatic breathing, segmental breathing, cough training, and inspiratory/expiratory muscle strengthening training using a Threshold IMT®/PEP® instrument. Resistance training: upper and lower extremity strengthening taught and told to repeatedly perform the resistance exercises at home.	Control: non-PR sample analysed: *n* = 12	Outcomes: VO_2max_; HR_max_; WR_peak_ Follow-up: no follow-up period Drop-outs: *n* = 2
Wallis et al. (2023) Single-cohort study Cycle CPET	*n* = 15 Age: 72.5 (69–80) years Gender = 13 M, 2F IPF diagnosed	Duration: 8 weeks Frequency: 2× per week Intensity: Training intensities were prescribed onto a chip and pin card, based on individual's physiological variables (AT and V˙O_2peak_) determined at CPET. This card was inserted into the cycle ergometer and participants instructed to pedal at a cadence of 60–65 r/min, according to a visible reading. Time: Sessions lasted for 30 min (week 1) or 40 min (weeks 2–8), starting with a 5-min warm-up at 0–5 watts. Type: After 5 min warm-up, the interval components began with 3 min, moderate intensity, at a work rate equal to 80% of that achieved at. This was followed by high intensity exercise, for 2 min at a work rate equal to 50% of the difference in work rate between AT and V˙O_2peak_. This 5-min interval was then repeated; 4 times (week 1) or 6 times (weeks 2–8), followed by a 5-min recovery period at 0–5 watts.	Control: Age and sex matched without IPF Sample analysed: *n* = 10	Outcomes: VO_2max_; WR_peak_; VE_peak_ Follow-up: no follow-up period Drop-outs: *n* = 4

M: male; F: female; LLEG: lower limb exercise group; ULEG: upper limb exercise group; VO_2max_: maximal volume of oxygen consumption; WR_peak_: peak work rate; HR_max_: maximum heart rate (BPM); VE_peak_: peak ventilation; AT: anaerobic threshold; HRR: heart rate reserve; CPET: cardiopulmonary exercise test; 6MWT: 6 min walk test; LAM: lymphangioleiomyomatosis; COPD: chronic obstructive pulmonary disease; Borg CR-10 scale: rate of perceived exertion 0–10 score; ILD: interstitial lung disease; HIIT: high intensity interval training.

### Participant characteristics

The 11 included studies consisted of 321 participants in total, of which 168 subjects were male. Two studies recruited single-sex participants only.^31,38^ Participant age ranged from 36 to 80 years with sample sizes ranging from 1^41^ to 52.^36^ Participants were categorised into interstitial lung disease-subtypes, including: IPF^33,35,37,39^ (*n* = 4), sarcoidosis^34,36,41^ (*n* = 3), lymphangioleiomyomatosis^31^ (*n* = 1), mixed-^32^ (*n* = 1), fibrosing-^40^ (*n* = 1), and dust-related interstitial lung disease^38^ (*n* = 1).

### Risk of bias

The overall critical appraisal skills programme quality of reporting score for the included single-cohort studies^31–37^ (*n* = 7) ranged from 8 to 12, with a mean score of 10 out of a possible maximum of 12 (Supplemental materials). The critical appraisal skills programme scores for the randomised controlled trials^38–40^ (*n* = 3) ranged from 7 to 10, with a mean score of 9 out of a possible maximum score of 11 (Supplemental materials). The most common reasons for a low critical appraisal skills programme score in the cohort studies were inadequate follow-up^31–35^ (*n* = 5) and inaccurate measure of exposure and outcome to minimise bias^34,37^ (*n* = 2). However, within the randomised controlled trials, low scores were most commonly found due to inadequate study methodology^39,40^ (*n* = 2).

### Cardiopulmonary exercise testing

All studies performed cardiopulmonary exercise tests using either a cycle ergometer^31,33^^36–39^^,41^ (*n* = 7) or a treadmill^32,34,35,40^ (*n* = 4). The CPET protocols, as described by authors, included: incremental^36,40,41^ (*n* = 3), both incremental ramp and constant work-rate exercise test^33,37^ (*n* = 2), ramp^39^ (*n* = 1), Balke^31^ (*n* = 1), modified Bruce^35^ (*n* = 1), modified Naughton^32^ (*n* = 1), step^35^ (*n* = 1), and symptom-limited and endurance cycle test^38^ (*n* = 1).

No study confirmed a plateau of oxygen consumption or performed supramaximal exercise bout to confirm a maximal effort; the indexes for potential identification of maximal effort reported were minute-ventilation/oxygen uptake slope^31,33,35,39,40^ (*n* = 5), rate of perceived exertion^31,35,36,38,41^ (*n* = 5), and respiratory exchange ratio^32,34,35,40^ (*n* = 4). Therefore, use of the term ‘maximal oxygen uptake’ herein does not necessarily reflect a true maximal effort, but reflects individual authors interpretation and reporting.^42^

### Exercise prescription

The quality of exercise reporting within the included studies was assessed using the aforementioned, 16-item internationally endorsed consensus on exercise reporting template to strengthen the assessments of the included studies^30^ (Supplemental materials). The overall consensus on exercise reporting template quality of exercise intervention reporting score for the included studies ranged from 6^36^ to 18,^33^ with a mean score of 14 out of a possible maximum of 19. The most common domains for a low consensus on exercise reporting template score within the included studies were inadequate description of participants baseline exercise experience^31,32^^34–41^ (e.g. beginner, intermediate, advanced, etc.) (*n* = 10), lack of detailed description for motivational strategies^31,32,36,39,40^ (*n* = 7), and poor description of non-exercise components^31,33,36,40,41^ (*n* = 5).

Exercise intervention duration ranged from 4 weeks^34^ to 4.5 years,^41^ with many of the included studies lasting 8–12 weeks.^33,35,38,39^ A total of *n* = 7 exercise programmes were supervised.^32,33,35,36^^38–40^ Four studies did not have a control group.^31,32,34,41^ The frequency of exercise ranged between 2× and 4× per week for all of the included studies. The initial time spent for each exercise session varied from 25^31^ to 90 min.^36,37^ A progressive duration of total exercise was performed in three studies,^31,38,40^ therefore by the end of the studies patients were performing at least a total of 45 min exercise sessions in all studies. No warm up duration was reported in seven studies.^31,33,34,^^36–38^^,40^ A warm up was performed in two studies^32,39^ for a duration of 5 to 10 min, which was not classed as part of the main exercise intervention.

The type of exercise differed within the included studies. Aerobic exercise was performed by walking,^32,38,40^ using a treadmill^32,34,35,40^ or cycling on an ergometer^33,^^36–38^ via methods of continuous^32,^^36–40^ or interval training.^33–35^ Some studies implemented upper limb and lower limb resistance exercises^34,^^36–40^ in combination with aerobic exercise,^31,35,^^37–40^ rather than focussing on one method alone. Breathing retraining and chest expansion exercises,^35,37^ flexibility,^36^ balance, and range of motion exercises^36^ and group training which included aerobics, water gymnastics, outdoor walking, medical yoga, and psychomotor physiotherapy^34^ were also implemented. Tailored pulmonary rehabilitation, within the included studies, was derived from guidelines related to chronic obstructive pulmonary diseases,^38,40^ respiratory disease,^39^ and cancer^33^; however seven studies^31,32,^^34–37^^,41^ did not use guidelines.

The cardiopulmonary exercise test-derived exercise prescription methods included: heart rate reserve^31,32,35^ (*n* = 3), maximum heart rate^34,36,40^ (*n* = 3), work rate^37–39^ (*n* = 3), anaerobic threshold and maximal oxygen uptake^33^ (*n* = 1), rate of perceived exertion^36^ (*n* = 1), and multiple methods^41^ (*n* = 1). Cardiac indexes were most commonly used to prescribe exercise within the six included studies.^31,32,^^34–36^^,40^ With relation to heart rate reserve derived exercise, a total of three studies used this index,^31,32,35^ however the intensities were different in each of these studies. For two steady-state studies, intensity was set at 65–75%^31^ and 70–80%^32^ heart rate reserve. For an interval training study,^35^ a 10-min warm-up at 50–70% heart rate reserve was conducted which was followed by five 3-min intervals of walking on a treadmill at 70–85% of the heart rate reserve, four 3-min walks at 50–70% of the heart rate reserve, and then a 10-min cool down at 50–70% of the heart rate reserve.^35^

A percentage of actual maximum heart rate was used in one study to prescribe treadmill intervals of 3-min at 85% maximum heart rate.^34^ The maximum heart rate equation (220 − age of the participant) was also utilised in one study for exercise prescription means^40^; this study used an initial low intensity of 50–60% maximum heart rate that progressed to a target of 30 min continuous, moderate intensity exercise at 64–76% maximum heart rate via a unique range of progressions in pulmonary rehabilitation design.^40^

Three studies utilised work rate indexes to define the intensity of their respective exercise programmes.^37–39^ The combination of anaerobic threshold and maximal oxygen uptake were used by one study to personalise exercise.^33^ Interval training was performed on an electromagnetically braked cycle-ergometer for 3 min at moderate intensity using 80% of work rate at the anaerobic threshold, followed by a 2-min block at severe intensity which was calculated by using work rate equal to midpoint between anaerobic threshold and peak oxygen consumption.^33^ Training intensity was progressed from 4× to 6× intervals after the first two sessions, or after the interim cardiopulmonary exercise test at week 4.^33^

### Synthesis of results

The main findings of the outcome measures are presented in Table 3. The most commonly reported cardiopulmonary exercise test outcomes within the included studies were maximal oxygen uptake^32–41^ (*n* = 11), maximum heart rate^31,32,^^34–36^^,38–41^ (*n* = 9), peak work rate^32,33,37–41^ (*n* = 7), and peak minute ventillation^32–34^^,38–41^ (*n* = 7). All the included studies that reported pre-to-post values for maximal oxygen uptake and peak work rate found improvements in these parameters (maximal oxygen uptake: 0.6^33^–44%^41^; peak work rate: 7^38^–32%^32^). Seven studies indicated statistically significant increases in peak work rate.^32,33,^^37–41^ Improvements in peak minute ventilation (5^34^–60%^41^) were found in six studies,^33,34,^^38–41^ with only one study indicating a decline in peak minute ventilation (−4%^32^). Maximum heart rate found variable results, whereby five studies^31,35,^^39–41^ increased (0.4^40^–6%^41^), while four studies^32,34,36,38^ decreased (0.7^34^–2.7%^32^). No adverse events were reported in any of the 11 included studies.

**Table 3.** Key CPET outcome measure findings.

Authors	VO_2max_ (mL/kg/min)	VE_peak_ (L/min)	HR_max_ (beats/min)	WR_peak_ (watts)
Arizono et al. (2014)	*Data reported as mL*/*min * EG: pre 651.7 ± 294.2 CG: pre 743.1 ± 255.0 EG: post 694.5 ± 284.0 CG: post 703.7 ± 237.3	Not reported	Not reported	EG: pre 60.8 ± 24.4 CG: pre 65.9 ± 17.1 EG: post 66.7 ± 26.2* CG: post 62.4 ± 19.3
Dale et al. (2014)	*Data reported as L*/*min* EG: pre 1.6 (0.5) vs CG: pre 1.6 (0.4); EG: post 1.6 (0.5) vs CG: post 1.6 (0.4) Difference within group EG: 0.1(0.1) vs CG: 0.1 (0.3) Mean difference (95% CI) between groups −0.01 (*p* = 0.882)	EG: pre 56 (21) vs CG: pre 55(12); EG: post 59 (24) vs CG: post 55 (13) Difference within group EG: 3(7) vs CG: 0.3 (11) Mean difference (95% CI) between groups 2.84 (−4.13, 9.80) (*p* = 0.412)	EG: pre 121 (22) vs CG: pre 123 (13); EG: post 120 (23) vs CG: post 126 (12) Difference within group EG: −0.5 (8) vs CG: 3 (11) Mean difference (95% CI) between groups −4 (−11, 3) (*p* = 0.238)	EG: pre 108 (41) vs CG: pre 110(33); EG: post 116 (43) vs CG: post 107 (30) Difference within group EG: 8(7) vs CG: −4 (12) Mean difference (95% CI) between groups 12 (5 ± 19) (*p* = 0.002*)
Vainshelboim et al. (2014)	Difference between groups EG 2.1 ± 2.3 vs CG 0.5 ± 2 Mean difference (95% CI) between groups 2.6 (1, 4.1*) (*p* = 0.002)	Difference between groups EG: 7 ± 6.9 vs CG: −4.5 ± 6.7 Mean difference (95% CI) between groups 11.6 (6.7, 16.5**) (*p *≤ 0.001)	EG: pre 74.8 ± 12 CG: pre 70.4 ± 15 (*p* = 0.373) EG: post +0.7 ± 9.1 CG: post +4.2 ± 9.3 Mean difference (95% CI) between groups 3.6 (–3.1, 10.2) (*p* = 0.283)	EG: 15.4 ± 9.8 vs CG: −6.7 ± 9.5 Difference between groups: 22.1 (15.1 ± 29.1) (*p *≤ 0.001**)
Keyser et al. (2015)	EG: pre 17.4 (5.5) vs EG: post 18.2 (5.0) Pre to Post difference +0.8 (*p* = 0.48)	EG: pre 15.3 (4.0) vs EG: post 14.7 (5.2) Difference −0.6 (*p* = 0.323)	EG: pre 148 (18.0) vs EG: post 144 (14.2) Difference −4 (*p* = 0.086)	EG: pre 129.5 ± 56.2 vs EG: post 170.5 ± 79.0 Difference +41.0 (*p* = 0.002*)
Grongstad et al. (2020)	EG: pre 24.6 ± 6.8 vs EG: post 25.8 ± 7.2 (*p* = 0.002)	EG: pre 75.4 ± 25 vs EG: post 79.3 ± 27 (*p* = 0.033)	EG: pre 153 ± 20 EG: post 152 ± 19 (*p* = 0.231)	Not reported
Herrera-Olivares et al. (2020)	EG: pre: 20.1 EG: post: 28.9 Change from Baseline to 4.5 years = +44%	EG: pre: 62 EG: post: 99 Change from Baseline to 4.5 years = +60%	EG: pre 99 vs EG: post 105 Rate of change (%) EG: 6	EG: pre 100 vs EG: post 124* Baseline to 4.5 years = 24%
Guber et al. (2021)	EG: 1.8 ± 2.3 (*p* = 0.002) increase pre-to-post	Not reported	EG: pre 137.8 ± 16 vs EG: post 134.2 ± 21 (*p* = 0.27)	Not reported
Essam et al. (2022)	LLEG: pre 17.5 (13.0–23.0) vs LLEG: post 22.0 (17.0–24.0) (*p* = 0.032*). ULEG: pre 10.5 (5.0–16.0). ULEG: post 13.5 (11.0–21.0) (*p* = 0.018*) Comparison between ULEG: post and LLEG: post (*p* = 0.075)	LLEG: pre 44.18 ± 11.50 vs LLEG: 54.67 ± 8.72 (*p* = 0.017*). ULEG: pre 31.81 ± 7.78 vs ULEG: 44.47 ± 7.00 (*p* = 0.003*). Comparison between ULEG: post and LLEG: post (*p* = 0.010*)	LLEG: pre 140 (122.25–144) vs LLEG: post 140.5 (119.5–147.0) (*p* = 0.715). ULEG: pre 122 (103.5–139.5) ULEG: post 130 (127–145.5) (*p* = 0.046*) Comparison between ULEG: post and LLEG: post (*p* = 0.705)	LLEG: pre 49.2 ± 14.48 vs LLEG: post 64.7 ± 11.51 (*p* = 0.009*) ULEG: pre 37.88 ± 21.62 vs ULEG: post 51.2 ± 20.36 (*p* = 0.006*) Comparison between ULEG: post and LLEG: post (*p* = 0.085)
Child et al. (2023)	EG: pre 25 ± 6 vs EG: post 26 ± 6; Rate of change (%) EG: 0.6 ± 1 (*p* = 0.21)	Not reported	EG: pre 159 ± 18 vs EG: post 163 ± 12 Rate of change (%) EG: 4 ± 7 (*p* = 0.08)	Not reported
Choi et al. (2023)	EG: pre 23 (20–25) vs EG: post 25 (23–28); Rate of change (%) EG: 10 (2–19) (*p* = 0.006) CG: pre 20 (15–23) vs CG: post 21 (16–25) Rate of change (%) EG: 7 (3–8) (*p* = 0.508)	Not reported	EG: pre 139 (127–158) vs EG: post 144 (132–160) Rate of change (%) EG: 4 (0–10) (*p* = 0.042) CG: pre 131 (106–150) vs CG: post 135(126–152) Rate of change (%) EG: 4 (−3, −7) (*p* = 0.270)	Not reported
Wallis et al. (2023)	EG: pre 11.95 (11.5–14.2) vs CG: pre 14.17 (13.2–19.8) (*p* = 0.160). EG: pre to post median difference +0.60 (−0.5 ± 1.2) (*p* = 0.328)	Pre to post difference +9% (2–19) (*p* = 0.05*)	Not reported	Difference +8% (6–16) (*p* = 0.010*)

Pre: pre-intervention; post: post-intervention; EG: exercise group; CG: control group; LLEG: lower limb exercise group; ULEG: upper limb exercise group; VO_2max_: maximal volume of oxygen consumption; WR_peak_: peak work rate; HR_max_: maximum heart rate (BPM); CI: confidence interval.

VO_2max_ scores reported as mL/kg/min, unless otherwise stated.

*p < 0.05; **p < 0.001.

## Discussion

This review highlights consistent improvements in maximum oxygen uptake, peak work rate, and peak minute ventilation following tailored, cardiopulmonary exercise test-derived pulmonary rehabilitation for people with interstitial lung disease. Peak work rate was the most consistently enhanced metric, with statistically significant gains across seven studies (+7^38^ to +32%^32^), supporting the physiological efficacy of tailored pulmonary rehabilitation. Peak ventilation improvements suggest enhanced ventilatory efficiency, while heterogeneous maximum heart rate responses (−3^32^ to +6%^41^) reflect variability potentially linked to cardiovascular conditioning,^22,43^ abnormal autonomic regulation,^22,43^ or inconsistencies in prescription methodology.

The exercise intensity that is applied for tailored pulmonary rehabilitation prescription is of paramount importance for optimising training outcomes.^22^ Many studies highlight that exercise intensities for tailored pulmonary rehabilitation should be based on the individuals cardiopulmonary exercise test measures,^33,36,44^ exercise performance,^31,36,45,46^ and preferences.^36^ Within the included studies, intensity was commonly set at fixed or ranged percentages of maximum heart rate, work rate, and cardiorespiratory performance values.

Exercise intensity was prescribed using heart rate recovery within four studies,^31,32,35^ however the intensities were markedly different. For the steady-state studies, intensity was set at 65–75%^31^ and 70–80%,^32^ and 50–70%^35^ to 70–85% for interval studies, showing a marked variation in exercise prescription methods with each interventional approach. All three studies found some benefit (+10%,^35^ +5%,^32^ +2.5%^31^) in maximal oxygen uptake; but also suggests room for improving exercise prescription methods. Moreover, intensity was also derived from the maximum heart rate equation (220 − age of the participant) in one study,^40^ with an initial low intensity exercise of 50–60% maximum heart rate that progressed to a target of 30 min continuous, moderate intensity exercise at 64–76% maximum heart rate via a unique range of progressions in pulmonary rehabilitation design.^40^ The results were far more promising than studies which utilised heart rate reserve to prescribe pulmonary rehabilitation; however, this must be taken with the consideration that the 220 − age equation is not a valid predictor of maximum heart rate^47^ and a younger patient sample with less advanced interstitial lung disease was used in this study.^40^

A ‘one size fits all’ approach, relying on predictive methods (220 − age) for maximum heart rate and estimated heart rate reserve to prescribe exercise appears to have some benefit as mean fitness increases have been shown in people with interstitial lung disease,^21^ aligning with similar evidence found in cardiology.^16^ However, it may be that fixed heart rate reserve ranges (e.g. UK guidelines: 40–70%) may not reflect true metabolic thresholds.^16^ A recent study found 54% of participants had ventilatory anaerobic thresholds outside this range^16^ and thus misaligned intensity can impair adherence and minimise benefit. Tailored approaches based on metabolic thresholds (e.g. gas exchange thresholds), may induce a more appropriate exercise stimulus which matches the persons capabilities, thus promoting adherence to pulmonary rehabilitation and the associated benefits.

When considering robustness of prescription, risk of bias appraisal indicated variable methodological quality, with cohort studies limited by follow-up (*n* = 5) and outcome measurement (*n* = 2), and bias in two randomised controlled trials linked to design limitations, emphasising the importance of transparent reporting to support clinical interpretation. Moreover, exercise reporting via the ‘Consensus on Exercise Reporting Template’ revealed frequent reporting gaps in baseline activity levels (*n* = 10) which could result in bias due to differences in the participants previous exercise levels, and possible motivation for participation in activity,^48^ as motivational strategies (*n* = 7) and non-exercise components (*n* = 5) were often omitted, hindering clinical translation.

Limitations in this review include significant heterogeneity in design, sex representation, pulmonary rehabilitation protocols, and interstitial lung disease subtypes, which precluded analysis and restricted generalisability. However, notable strengths include this being the first systematic review to evaluate the role of cardiopulmonary exercise test-derived, tailored pulmonary rehabilitation in people with interstitial lung disease, synthesising data from 11 studies involving 321 participants; an encouraging yield for an emerging field. Robust appraisal tools strengthen the methodological rigour^30^ and highlights pathways for clinical implementation.

In summary, tailored pulmonary rehabilitation, informed by CPET, shows promise in enhancing exercise capacity and ventilatory efficiency for interstitial lung diseases. Consistent improvements in maximum oxygen uptake, peak work rate, and peak ventilation support its clinical utility, while variability in cardiac-based responses underscores need for more precise prescription methods. Future research should compare tailored pulmonary rehabilitation with standard care, evaluate alternative cardiopulmonary exercise test prescription indexes (such as the gas exchange threshold), and adopt transparent, standardised reporting for clinical translation.

## Clinical messages

- Tailored pulmonary rehabilitation may improve exercise outcomes such as peak oxygen uptake and peak work rate in people with interstitial lung disease.
- All seven studies assessing peak work rate reported statistically significant improvements, supporting its use as a primary outcome measure for rehabilitation effectiveness.
- No serious adverse events were reported across the included studies, confirming the safety of structured rehabilitation programmes for this population.
- Exercise prescription methods varied between studies; future research should prioritise standardised protocols and consistent reporting to enhance clinical applicability.
